# Astragalus polysaccharide enhances maternal mucosal immunity against PEDV

**DOI:** 10.1128/msphere.00777-24

**Published:** 2024-11-14

**Authors:** Jianli Tang, Shuaiyong Wang, Jianmei Tang, Jinming Li

**Affiliations:** 1 Carter Immunology Center, University of Virginia, Charlottesville, Virginia, USA; 2 College of Agriculture and Forestry Science, Linyi University, Linyi, China; 3 Department of Technology Service, Hebei Qinkang Biotechnology Co., Ltd., Tangshan, China; Instituto de Biotecnologia/UNAM, Cuernavaca, Morelos, Mexico

**Keywords:** immune response, PEDV, mucosal vaccines

## Abstract

Porcine epidemic diarrhea virus (PEDV), the major causative pathogen of porcine epidemic diarrhea, poses a severe threat to the swine industry, particularly affecting neonatal piglets. Maternal milk-derived IgA antibody is crucial for protecting piglets from PEDV infection. Despite the effectiveness of current intramuscularly administered PEDV vaccines in inducing strong systemic immune responses, their ability to generate high levels of maternal milk IgA is limited. This study explores the potential of Astragalus polysaccharide (APS) to enhance PEDV vaccine efficacy, specifically focusing on maternal milk IgA levels. We first evaluated anti-PEDV antibody levels in the blood and colostrum of sows vaccinated with PEDV or subjected to feedback feeding. Our results indicated that while vaccination induced robust serum PEDV-specific IgG and IgA, milk IgA levels were lower compared to the feedback group. To address this limitation, APS was administered orally to sows before PEDV vaccination. APS supplementation significantly increased both serum and milk PEDV-specific IgA levels and enhanced cellular immune responses, as evidenced by elevated cytokine levels. Further analysis demonstrated that APS improved intestinal immune function and homeostasis in piglets. Overall, APS supplementation proved to be an effective immune booster, enhancing PEDV vaccine-induced mucosal immunity and providing a promising strategy for improving maternal immunity and piglet protection against PEDV.

**IMPORTANCE:**

This study highlights the limitations of current porcine epidemic diarrhea virus (PEDV) vaccines in inducing sufficient maternal milk IgA, which is crucial for protecting neonatal piglets. By supplementing Astragalus polysaccharide (APS) into the vaccination regimen, we demonstrated a significant enhancement in milk PEDV-specific IgA levels, as well as improved cellular immune responses. APS also bolstered intestinal immune function and homeostasis in piglets. These findings suggest that APS supplementation could serve as an immune booster to enhance maternal immunity, offering a promising approach to better protect piglets against PEDV.

## OBSERVATION

Porcine epidemic diarrhea, caused by the porcine epidemic diarrhea virus (PEDV), remains an enormous threat to the swine industry (1). While PEDV infects pigs of all ages, it predominantly causes severe symptoms and high mortality in neonatal suckling piglets (2). The virus primarily targets the intestinal tract, where it infects and replicates in the villous epithelial cells of the small intestine, jejunum, and the epithelial cells of the cecum and colon (3). PEDV is predominantly transmitted via the fecal-oral route, through contact with infected pigs or feces-contaminated materials (4). Thus, developing therapeutics or vaccines that specifically target the intestine is a promising strategy for controlling the PEDV pandemic.

In neonatal piglets, the immune system is not yet fully developed. Consequently, maternal immunity, particularly milk-derived IgA antibodies, plays a pivotal role in protecting piglets from PEDV infection. PEDV-specific IgA binds to the virus at the mucosal surface, thereby preventing its attachment to and penetration of intestinal epithelial cells (5). Maternal PEDV-specific IgA, induced by vaccination before farrowing, is transferred into the milk via the gut-mammary gland-secretory immunoglobulin A axis (6). Thus, inducing robust maternal intestinal mucosal immunity is considered critical for safeguarding piglets against PEDV infection.

The current strategy of combining live-attenuated and inactivated vaccines administered through intramuscular immunization is regarded as an effective approach to controlling the PEDV pandemic by inducing robust humoral and cellular immunity (7). Additionally, administering feedback (introducing the intestines of PEDV-infected piglets) is commonly practiced to induce herd immunity among all sows in the affected herd (8). Nevertheless, there is ongoing debate about whether intramuscular PEDV vaccination can effectively stimulate high levels of IgA in milk. Therefore, further investigation is required to reveal the maternal PEDV vaccine-induced mucosal immunity in the intestine.

Growing evidence suggests that Astragalus polysaccharide (APS) can profoundly affect the immune system. A recent study demonstrated that APS, as an adjuvant, greatly boosted the efficacy of the influenza vaccine, as evidenced by enhanced neutralizing antibody levels, a higher frequency of specific CD8^+^ cells, and an increased survival rate in influenza-infected mice. Furthermore, APS was shown to alleviate alveolar damage in lung tissue and maintain intestinal homeostasis (9). Another study further underscores the immunomodulatory effects of APS in enhancing both humoral and cellular immune responses in pigs vaccinated against foot-and-mouth disease virus (10). Moreover, intranasal vaccination with APS conferred robust protective mucosal immunity against *Helicobacter pylori* infection (11). However, whether APS administration induces or enhances mucosal immunity against PEDV in vaccinated sows has yet to be investigated.

In this study, we first investigated the anti-PEDV antibody levels in the blood and colostrum of sows that were either vaccinated or subjected to feedback. Subsequently, APS was orally administered to the sows prior to PEDV vaccination. Our findings showed that PEDV vaccine effectively induced strong circulating humoral and cellular immune responses but induced relatively suboptimal milk IgA levels compared to the feedback group. Moreover, APS proved to be an effective immune booster, considerably enhancing the maternal IgA level induced by the PEDV vaccine.

### Vaccination elicited relatively weak milk PEDV-specific IgA than feedback

**Fig 1:**
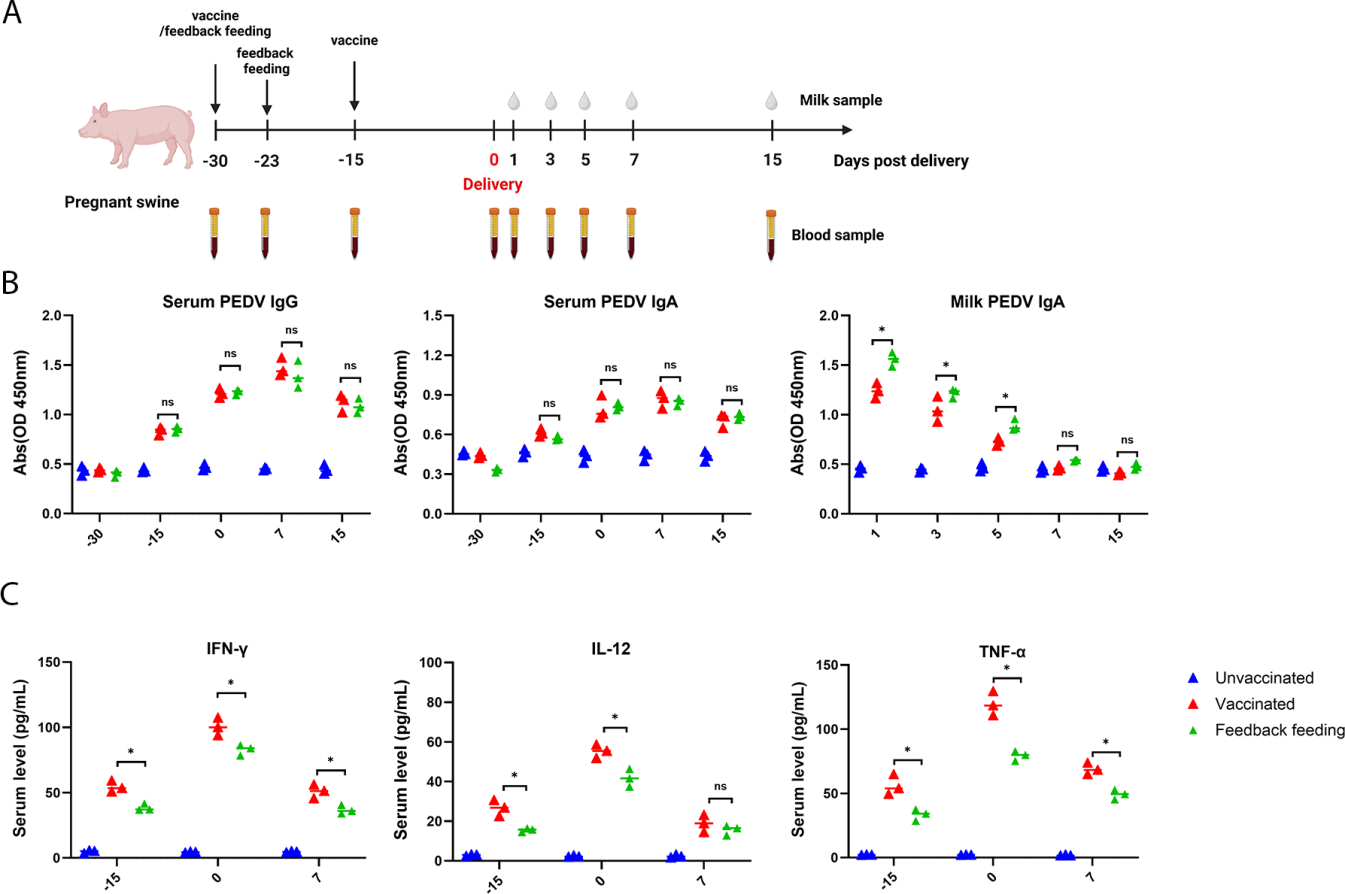
Humoral and cellular immune responses in vaccinated and feedback-feeding sows. (**A**) Schematic flow diagram of sow immunization procedure (*n* = 3 for unvaccinated, *n* = 3 for vaccinated, and *n* = 3 for feedback-feeding sows). (**B**) Serum IgG, IgA, and milk IgA levels of sows were measured using ELISA. (**C**) Cytokine levels in blood were quantified by ELISA. All data are presented as means ± SEM, and statistical differences were assessed using one-way ANOVA. Statistical significance is indicated as follows: ns, not significant (*P* > 0.05); * (*P* < 0.05).

The experimental process was carried out as illustrated in Fig. 1A. We performed the enzyme-linked immunosorbent assay (ELISA) to assess and compare the serum antibodies induced by the PEDV vaccine and feedback in pregnant sows. As shown in Fig. 1B, vaccination elicited strong PEDV-specific serum IgG levels, which were comparable to those in the feedback-feeding group from days postdelivery (dpd) −15 to dpd 15. Meanwhile, the serum PEDV-specific secretory immunoglobulin A (IgA) levels were also comparable between the vaccination and feedback-feeding groups. We further determined the PEDV-specific IgA levels in milk after farrowing. Compared to unvaccinated sows, both vaccination and feedback feeding induced high levels of IgA in milk. Importantly, the IgA level in the feedback-feeding group was significantly higher than in the vaccination group from dpd 1 to dpd 3 (Fig. 1B).

Furthermore, ELISA was performed to determine the levels of cytokines IFN-γ, IL-12, and TNF-α to assess the cellular immune response and inflammatory response (Fig. 1C). Compared with the feedback-feeding group, IFN-γ, IL-12, and TNF-α were significantly enhanced in the vaccination group at dpd −15 and dpd 0. In addition, vaccination induced higher IFN-γ and TNF-α compared to the feedback-feeding group at dpd 7.

Our results indicate that while vaccination induces a robust cellular immune response and comparable levels of PEDV-specific IgA and IgG in the blood, it elicits relatively lower IgA levels in milk compared to the feedback-feeding group in pregnant sows.

### Oral administration of APS enhances the maternal milk PEDV-specific IGA after vaccination

**Fig 2:**
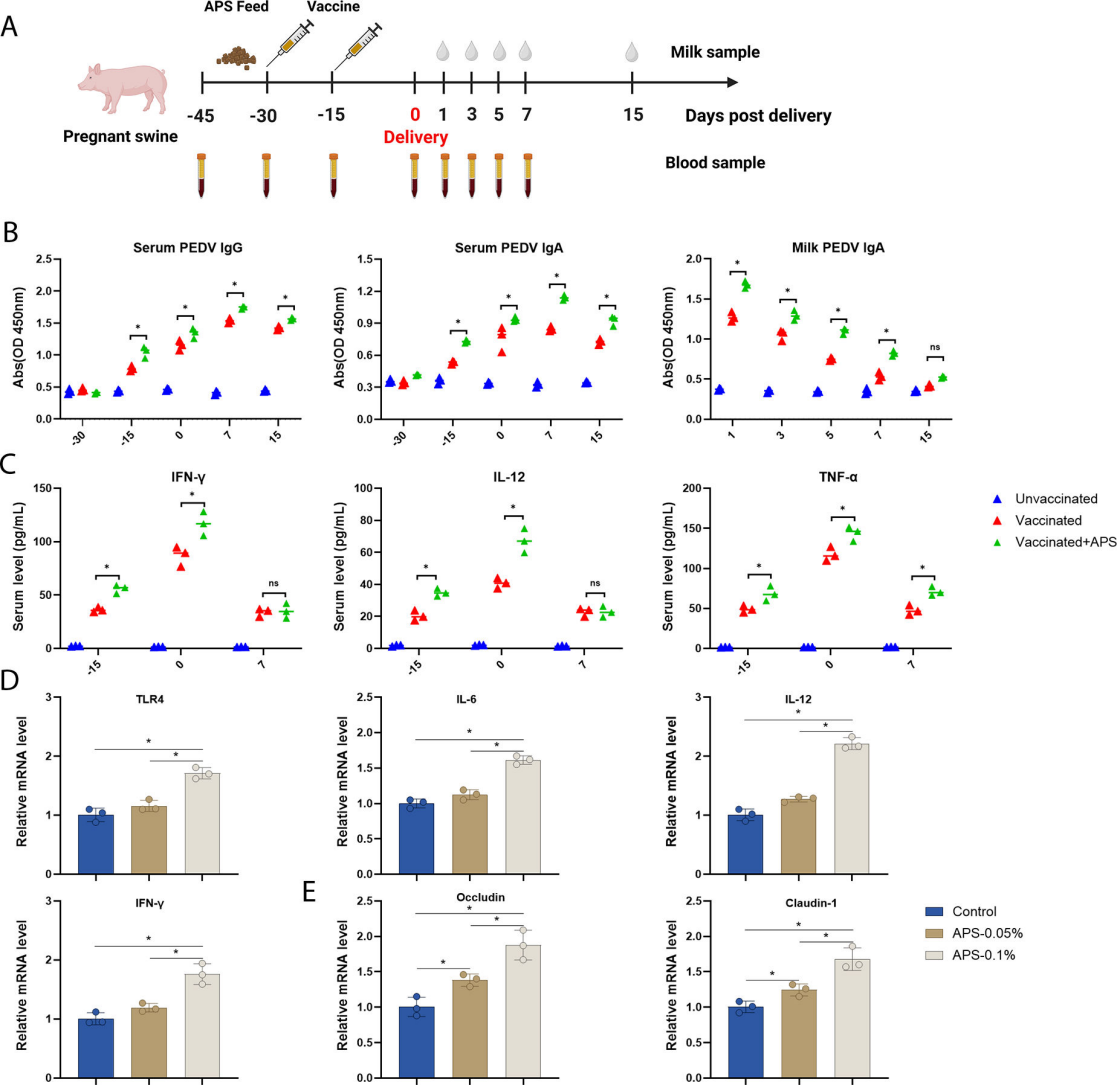
APS supplementation boosts both humoral and cellular immune responses in sows inoculated with the PEDV vaccine. (**A**) Schematic flow diagram of sow immunization procedure (*n* = 3 for unvaccinated sows, *n* = 3 for vaccinated, and *n* = 3 for APS plus vaccine). (**B**) Serum IgG, IgA, and milk IgA levels of sows were measured using ELISA. (**C**) Blood cytokine levels of sows were quantified by ELISA. (**D and E**) Intestinal expression levels of IL-12, IFN-γ, IL-6, TLR4 occludin, and claudin-1 in piglets were determined using RT-qPCR. All data are presented as means ± SEM, and statistical differences were assessed using one-way ANOVA. Statistical significance is indicated as follows: ns, not significant (*P* > 0.05); * (*P* < 0.05).

To examine the immune-enhancing effect of APS on the PEDV vaccine, we conducted the experimental procedure as shown in Fig. 2A. ELISA was performed to measure serum and milk antibodies. Our results revealed that PEDV vaccination plus oral APS administration significantly enhanced PEDV-specific serum IgG and IgA levels compared to vaccination alone (Fig. 2B). Furthermore, significantly higher levels of milk PEDV-specific IgA were induced in the group receiving PEDV vaccination combined with oral APS administration. The cytokines IFN-γ, IL-12, and TNF-α were significantly upregulated in the APS-supplemented group at dpd −15 and dpd 0. Moreover, TNF-α levels were greatly elevated in the APS-supplemented group at dpd 7 compared to the vaccination-only group (Fig. 2C). We further investigated the role of APS in boosting the innate immune response in piglets. As shown in Fig. 2D, 0.1% APS supplementation significantly increased the expression levels of TLR4, IL-6, IL-12, and IFN-γ compared to the control group. Recent studies have suggested that APS promotes intestinal development and homeostasis (12). Therefore, we examined the expression of occludin and claudin-1, key markers of intestinal barrier function. Both occludin and claudin-1 were significantly upregulated in the 0.1% APS supplementation group compared to the control (Fig. 2E). Overall, APS supplementation enhances the cellular immune response and increases PEDV-specific antibodies in the blood and milk of sows. Additionally, APS supplementation supports intestinal homeostasis and strengthens intestinal immune function.

### Conclusion

For piglets, the protective efficacy of the PEDV vaccine primarily depends on maternal secretory IgA (6). Compared to intramuscular injection, oral inoculation elicits a more robust anti-PEDV immune response (13). Since most commercial PEDV vaccines are administered via intramuscular injection, their ability to induce maternal milk IgA in sows requires further investigation. In this study, we found that the PEDV vaccine induced relatively lower levels of maternal milk IgA compared to feedback sows. Nevertheless, vaccination generated stronger serum PEDV-specific IgG and IgA and a more robust cellular immune response, as evidenced by the enhanced cytokine levels. Due to the low pH and high digestive capacity of the stomach, oral vaccination encounters significant challenges (14). Growing evidence indicates that APS possesses immune-enhancing properties (15). Therefore, we supplemented sows with APS prior to vaccination. Our results demonstrated that APS significantly increased IgA levels in milk and enhanced both humoral and cellular immune responses in the blood against PEDV. In piglets, APS supplementation also promoted intestinal homeostasis and strengthened local innate immunity. In conclusion, APS serves as an effective immune booster for PEDV vaccination.

## AFTER EPUB

*[This article was published on 14 November 2024 with errors in the text, figure legends, and supplemental material. The errors were corrected in the current version, posted on 4 December 2024.]*

## SUPPLEMENTAL MATERIAL

Supplemental material msphere.00777-24-s0001.docx Supplemental text and table.

ASM does not own the copyrights to Supplemental Material that may be linked to, or accessed through, an article. The authors have granted ASM a non-exclusive, world-wide license to publish the Supplemental Material files. Please contact the corresponding author directly for reuse.

## References

[1] Jung K, Saif LJ, Wang Q. 2020. Porcine epidemic diarrhea virus (PEDV): an update on etiology, transmission, pathogenesis, and prevention and control. Virus Res 286:198045. doi:10.1016/j.virusres.2020.198045 32502552 PMC7266596

[2] Zhang H, Zou C, Peng O, Ashraf U, Xu Q, Gong L, Fan B, Zhang Y, Xu Z, Xue C, Wei X, Zhou Q, Tian X, Shen H, Li B, Zhang X, Cao Y. 2023. Global dynamics of porcine enteric coronavirus PEDV epidemiology, evolution, and transmission. Mol Biol Evol 40:msad052. doi:10.1093/molbev/msad052 36869744 PMC10027654

[3] Fan B, Zhou J, Zhao Y, Zhu X, Zhu M, Peng Q, Li J, Chang X, Shi D, Yin J, Guo R, Li Y, He K, Fan H, Li B. 2023. Identification of cell types and transcriptome landscapes of porcine epidemic diarrhea virus–infected porcine small intestine using single-cell RNA sequencing. J Immunol 210:271–282. doi:10.4049/jimmunol.2101216 36548460

[4] Kim Y, Yang M, Goyal SM, Cheeran M-J, Torremorell M. 2017. Evaluation of biosecurity measures to prevent indirect transmission of porcine epidemic diarrhea virus. BMC Vet Res 13:89. doi:10.1186/s12917-017-1017-4 28381304 PMC5382501

[5] Suda Y, Miyazaki A, Miyazawa K, Shibahara T, Ohashi S. 2021. Systemic and intestinal porcine epidemic diarrhea virus-specific antibody response and distribution of antibody-secreting cells in experimentally infected conventional pigs. Vet Res 52:2. doi:10.1186/s13567-020-00880-z 33397461 PMC7780908

[6] Chattha KS, Roth JA, Saif LJ. 2015. Strategies for design and application of enteric viral vaccines. Annu Rev Anim Biosci 3:375–395. doi:10.1146/annurev-animal-022114-111038 25387111

[7] Kong F, Jia H, Xiao Q, Fang L, Wang Q. 2023. Prevention and control of swine enteric coronaviruses in China: a review of vaccine development and application. Vaccines (Basel) 12:11. doi:10.3390/vaccines12010011 38276670 PMC10820180

[8] Yamagami T, Miyama T, Toyomaki H, Sekiguchi S, Sasaki Y, Sueyoshi M, Makita K. 2021. Analysis of the effect of feedback feeding on the farm-level occurrence of porcine epidemic diarrhea in Kagoshima and Miyazaki Prefectures, Japan. J Vet Med Sci 83:1772–1781. doi:10.1292/jvms.21-0343 34615808 PMC8636866

[9] Wan C, Yan S, Lu R, Zhu C, Yang Y, Wu X, Yu Z, Jiang M, Peng W, Song W, Wu H, Fang B, He Y. 2024. Astragalus polysaccharide improves immunogenicity of influenza vaccine as well as modulate gut microbiota in BALB/c mice. Microb Pathog 195:106893. doi:10.1016/j.micpath.2024.106893 39197333

[10] Li J, Zhong Y, Li H, Zhang N, Ma W, Cheng G, Liu F, Liu F, Xu J. 2011. Enhancement of Astragalus polysaccharide on the immune responses in pigs inoculated with foot-and-mouth disease virus vaccine. Int J Biol Macromol 49:362–368. doi:10.1016/j.ijbiomac.2011.05.015 21640133

[11] Liu C, Luo J, Xue RY, Guo L, Nie L, Li S, Ji L, Ma CJ, Chen DQ, Miao K, Zou QM, Li HB. 2019. The mucosal adjuvant effect of plant polysaccharides for induction of protective immunity against Helicobacter pylori infection. Vaccine (Auckl) 37:1053–1061. doi:10.1016/j.vaccine.2018.12.066 30665774

[12] Yang SB, Qin YJ, Ma X, Luan WM, Sun P, Ju AQ, Duan AY, Zhang YN, Zhao DH. 2021. Effects of in ovo injection of Astragalus polysaccharide on the intestinal development and mucosal immunity in broiler chickens. Front Vet Sci 8:738816. doi:10.3389/fvets.2021.738816 34527718 PMC8435677

[13] Zhou Y, Chen C, Chen Y, Liu Z, Zheng J, Wang T, Luo H, Liu Y, Shan Y, Fang W, Li X. 2019. Effect of route of inoculation on innate and adaptive immune responses to porcine epidemic diarrhea virus infection in suckling pigs. Vet Microbiol 228:83–92. doi:10.1016/j.vetmic.2018.11.019 30593385 PMC7173071

[14] Wen Z, Xu Z, Zhou Q, Li W, Wu Y, Du Y, Chen L, Zhang Y, Xue C, Cao Y. 2018. Oral administration of coated PEDV-loaded microspheres elicited PEDV-specific immunity in weaned piglets. Vaccine (Auckl) 36:6803–6809. doi:10.1016/j.vaccine.2018.09.014 30243502

[15] Yin X, Chen L, Liu Y, Yang J, Ma C, Yao Z, Yang L, Wei L, Li M. 2010. Enhancement of the innate immune response of bladder epithelial cells by Astragalus polysaccharides through upregulation of TLR4 expression. Biochem Biophys Res Commun 397:232–238. doi:10.1016/j.bbrc.2010.05.090 20546703

