# Supplementary material for: Astragalus polysaccharide enhances maternal mucosal immunity against PEDV
Source: mSphere. 2024 Nov 14;9(12):e00777-24. doi: 10.1128/msphere.00777-24 (PMC11656797; doi:10.1128/msphere.00777-24)
Supplement: Supplemental material — Supplemental text and table. [file msphere.00777-24-s0001.docx]

**MATERIALS AND METHODS**

**Feedback materials preparation**

Feedback was made as previously reported. PEDV-infected piglets were confirmed by quantitative real-time PCR (RT-qPCR). PEDV RT-qPCR kit was purchased from MEDIAN Diagnostics (NS-PED-31). PEDV-positive piglets were euthanized and the intestine was homogenized followed by adding 5 L distilled water, 5 mL 4% gentamicin, and 200 g milk replacer. Sows were orally administered 100 mL homogenate.

**Immunization and sample collection**

Protocols in this study were approved by the Ethics Committee of Linyi University. Hebei Qinkang Co., Ltd helped to perform the pig experiments, all sows and piglets were tested negative for PEDV using virus-specific RT-qPCR and serum neutralization tests. Nine pregnant sows were randomly divided into three groups: (1) feedback feeding group (n = 3); (2) PEDV commercial vaccine (n = 3); and (3) PBS control group (n = 3). For immunization, sows were immunized with the commercial live-attenuated vaccine or fed with feedback materials 30 days before farrowing and were given feedback materials 7 days later or given an inactivated vaccine 15 days before delivery. Serum samples were collected from sows at -30, -15, 0, 7, 15 days post-delivery. Nine pregnant sows were randomly divided into three groups: (1) vaccination group (n = 3); (2) APS plus vaccine (n = 3); and (3) PBS control group (n = 3). APS plus vaccine group sows were fed with APS for 15 days before immunization. For immunization, sows were immunized with the commercial live-attenuated vaccine 30 days before farrowing and were given an inactivated vaccine 15 days before delivery. Serum samples were collected from sows at -30, -15, 0, 7, 15 days post-delivery. Serum samples were inactivated at 56 °C for 30 min and stored at -80 °C. Moreover, milk samples were collected from the sows at 1, 3, 5, 7, and 15 days post-delivery and were stored at -80°C.

APS was purchased from DBN Group (Beijing, China). Six piglets at 7 days old were fed with APS-supplemented water (0.05 %, 0.10 %), and a control group was fed with non-APS-supplemented water (0.00 %) for 14 days. Next, the piglets were vaccinated with the PEDV commercial vaccine, piglets were euthanized 14 days post-vaccination. The intestinal were collected and stored at -80 °C.

**Assessment of immune gene expression in the intestine**

RNAiso Plus (9108, TaKaRa) was used to extract RNA from intestine tissues, followed by reverse transcription to synthesize cDNA using 5 x RT master mix (RR036Q, TaKaRa). RT-qPCR assays were performed to determine the expression levels of target genes. All primers used in the RT-qPCR were listed in Table .1.

**Table 1.** Primer sequences of target genes.

| Gene | Primer sequences (5`-3`) |
| --- | --- |
| TLR4 | F: TTGAACAGTTCCGGATAGCACA  R: GGCTTCTAGACCACGCAAATTC |
| IL-6 | F: CCAGGAACCCAGCTATGAAC  R: CTGCACAGCCTCGACATT |
| IL-12 | F: CCGTCAGCAACACACTTC  R: TTCAGAGCCTGCATCAGC |
| TNF-α | F: AACCTCAGATAAGCCCGTCG  R: ACCACCAGCTGGTTGTCTTT |
| Occludin | F: CAGTGGTAACTTGGAGGCGT  R: CCGTCGTGTAGTCTGTCTC |
| Caludin-1 | F: GGACAAAACCGTGTGGGAAC  R: ACATGAAAATGGCTTCCCTCC |
| GAPDH | F: CCTTCCGTGTCCCTACTGCCAAC  R: GACGCCTGCTTCACCACCTTCT |

**Enzyme-linked immunosorbent Assay (ELISA) for antibody and cytokines detection**

The AsurDxTM Porcine Epidemic Diarrhea Virus (PEDV) IgA antibody test kit (10056-05) and AsurDx™ Porcine Epidemic Diarrhea Virus (PEDV) IgG antibody test kit (10055-05) were purchased from BioStone Animal Health (Guangzhou, China). Porcine IL12 ELISA kit (RAB0868), Porcine IFN γ ELISA kit (RAB0226), and Porcine Tumor Necrosis Factor α ELISA kit (RAB0478) were purchased from Sigma (MA, USA). ELISA was performed according to the manufacturer's protocol. Briefly, 96-well microplates precoated with the specific antigen were added with diluted samples and incubated at 37 C for 1 h. After three times washing, the plates were added with horseradish peroxidase (HRP) conjugated secondary antibody and incubated at 37 C for 1 h. After three times rinse, tetramethylbenzidine substrate solution was added to each well, and the reaction was stopped by adding the stop solution after 15 min. The optical density (OD) of the wells was read at a wavelength of 450 nm.
